# The Digital Determinants of Health: A Guide for Competency Development in Digital Care Delivery for Health Professions Trainees

**DOI:** 10.2196/54173

**Published:** 2024-08-29

**Authors:** Katharine Lawrence, Defne L Levine

**Affiliations:** 1 Department of Population Health, New York University Grossman School of Medicine, 227 East 30th Street 6th Floor, New York, NY, 10016, United States, 1 6465012684

**Keywords:** digital health, digital determinants of health, digital health competencies, medical education curriculum, competency development, digital health education, training competencies, digital health skills, digital care delivery, health professions training

## Abstract

Health care delivery is undergoing an accelerated period of digital transformation, spurred in part by the COVID-19 pandemic and the use of “virtual-first” care delivery models such as telemedicine. Medical education has responded to this shift with calls for improved digital health training, but there is as yet no universal understanding of the needed competencies, domains, and best practices for teaching these skills. In this paper, we argue that a “digital determinants of health” (DDoH) framework for understanding the intersections of health outcomes, technology, and training is critical to the development of comprehensive digital health competencies in medical education. Much like current social determinants of health models, the DDoH framework can be integrated into undergraduate, graduate, and professional education to guide training interventions as well as competency development and evaluation. We provide possible approaches to integrating this framework into training programs and explore priorities for future research in digitally-competent medical education.

## Introduction

The COVID-19 pandemic heralded a transformation in care delivery to virtual services and digital technologies such as telemedicine, remote patient monitoring, and asynchronous patient portal communications. This transition, coupled with the growing field of “Big Data” informatics and generative artificial intelligence (“GenAI”), has reinvigorated enthusiasm in the “digital transformation” of health care [1] and the use of novel digital technologies to provide personalized, convenient, and comprehensive care for all. It has also resulted in calls to improve the “digital health competencies” of clinicians, to help both current health care providers and trainees meet this transformative moment in care delivery [2-4].

Digital health tools—which include a wide range of “virtual” technologies such as telemedicine, remote sensors, and wearables, as well as medical “apps” and eHealth and mobile health tools, digitized health record and communications platforms (electronic health records [EHRs] and patient portals), clinical decision support systems, and personalized and predictive modeling technologies [5]—have been progressively integrated into mechanisms of care delivery over the last decade, with growing support from both patients and clinicians [3,6,7]. Patient empowerment and self-management are factors that contribute to patient use of digital health [8]. In the United States, 93% of physicians believe digital health tools are an advantage for patient care, with the majority citing a desire to provide competent remote care to patients as a significant motivator to adopt digital tools [6].

Among medical trainees, sentiments around use of digital health technologies are similar, with these technologies increasingly becoming inseparable from medical training [9,10]. This trend accelerated during the COVID-19 pandemic, as resident clinics pivoted to telemedicine and training shifted to virtual conferences, e-learning modules, and telesimulation [11,12]; nursing and other allied health professions saw similar shifts in their own education and care delivery experiences [13,14]. This dramatically shifted environment has created an appetite for both learning and teaching digital health skills among medical trainees, while also exposing gaps in current approaches to curricular development, implementation, and evaluation [3,4,15-17]. At the same time, there is growing recognition of the equity risks associated with digital health technology [18,19], particularly as the use of these tools was expanded during the pandemic and disparities in access and proficiency widened existing health care inequalities [18,20-22]. This reality underscores the need to cultivate a health care workforce that is both technically *and* culturally competent, as well as to better integrate health equity efforts in clinical training.

This paper explores the current state of digital health education and training competencies among medical and allied health professions through a brief narrative review and identifies key limitations in these approaches. We then offer a novel framework—the digital determinants of health (DDoH)—that can help unify and direct ongoing competency development and evaluation efforts. The DDoH framework can also ensure that key equity considerations of digital health are incorporated into trainee competencies, thereby helping reduce disparities associated with this these technologies’ use.

## Current Digital Health Education and Training

At its core, the challenge of teaching digital health competencies to medical trainees lies in the lack of consensus regarding *what* those competencies are and *how* they should be taught. While several major medical organizations in the United States and internationally have released statements [23-25] regarding some element of digital health competencies at undergraduate, graduate, and professional continuing medical education levels, significant heterogeneity exists in these organizations’ definitions, areas of focus, and evaluation tools and metrics (Table 1). A brief narrative review of the current medical literature on the topic of “digital health training” reveals both vagueness and variability in the definition of “digital health,” with overrepresentation of language from biomedical informatics, health information technology, and telemedicine. Often, only general recommendations for training competency domains (eg, patient safety and medical knowledge) are offered, rather than any specific competencies. Existing instruments to measure competencies often focus on specific use cases (eg, EHR proficiency) rather than the broad-scope digital health tools and services that exist today [2]. Many instruments are not validated, being either adapted from previously developed tools or newly designed to meet the changing technological landscape and educational needs [2].

**Table 1.** Brief narrative review of digital health technology definitions, domains, competencies, and skills.

Source	Digital health definition	Main domains or technologies	Competencies and skills
Accreditation Council for Graduate Medical Education	Not defined: reviewed competencies relevant to digital health but not explicitly digital health specific	Specific domains and technologies are not recognized in core competencies	Broad competencies encapsulate patient care, medical knowledge, practice-based learning and improvement, systems-based practice, interpersonal and communication, and professionalism. None are specific digital health competencies [26].
American Medical Association (AMA) [27]	Definition: “Digital health encompasses a broad scope of tools that can improve health care, enable lifestyle change and create operational efficiencies” [27]	Digital solutions: telemedicine and telehealth, mHealth^a^, wearables, remote monitoring, and apps	While specific competencies are not outlined, the AMA has been studying, since 2016, physicians’ motivations for using digital clinical tools.
Association of American Medical Colleges (AAMC) [23]	Not defined	Telehealth competencies across 6 domains and 3 tiers	Domains: “Patient safety and appropriate use of telehealth, access and equity in telehealth, communication via telehealth, data collection and assessment via telehealth, technology for telehealth, ethical practices and legal requirements for telehealth.” Competency tiers: “entry to residency or recent medical school graduate, entry to practice or recent residency graduate, experienced faculty physician or three to five years post-residency” [23].
Centers for Disease Control and Prevention (CDC)	Definition: “the systematic application of information and communications technologies, computer science, and data to support informed decision-making by individuals, the health workforce, and health systems, to strengthen resilience to disease and improve health and wellness” [28]	Outlines key competencies for public health professionals [29]	The CDC uses the 10 essential public health services to guide its competencies. These 10 services do not include digital health–specific competencies [30].
The Standing Committee of European Doctors (CPME) [25]	Not defined: digital competency web page focuses on digital health literacy of health professionals	Calls on members to support investing in eHealth solutions to improve patient care and expand digital health literacy	The CPME does not list specific digital competencies in this statement but outlines the importance of digital competencies, given the way digital health is transforming medicine and health care.
Royal Australasian College of Physicians (RACP); Scott et al [4]	Definition: digital health encompasses digital systems integrated in health care and “extends beyond electronic storage, retrieval or transmission of data to the active use of these data in quality improvement, service redesign and knowledge development” [4]	Digital systems: EMRs^b^, e-ordering, e-prescribing, virtual care, e-messaging, e-consults, clinical decision support, mHealth, remote patient monitoring, and artificial intelligence	11 foundational digital competencies in knowledge and understanding outlined over 3 digital health capability horizons: “Horizon 1: Embedding safe, ethical, and effective use of systems if electronic records Horizon 2: Integrating new technologies and ways of working Horizon 3: Digital health transformation” [4].
World Health Organization (WHO)	Definition: within global strategies for digital health, the WHO defines digital health as “the field of knowledge and practice associated with the development and use of digital technologies to improve health” [31]	Domains encompassed: eHealth, advanced computing, big data, and artificial intelligence	The WHO proposes in their global strategy on digital health (2020‐2025) to identify core digital health literacy competencies in short term for training of health professionals and ensure that digital health competencies are integrated into education [31].
Longhini et al [17]	Not defined: uses the WHO definition of digital health interventions, “discrete function of digital technology to achieve health care sector objectives” [32]	Digital health competencies including terms related to digital literacy, health informatics, and eHealth	Four main categories of digital health competencies identified (with subcategories): Category 1: “self-rated competencies” Subcategories: “digital literacy,” “eHealth literacy,” “patient-oriented competencies,” and “process of care-oriented competencies.” Category 2: “psychological and emotional aspects towards digital technologies” Subcategories: “attitudes and beliefs,” “confidence,” and “awareness” Category 3: “use of digital technologies” Subcategory: “general use of digital technologies” Category 4: “knowledge about digital technologies”
Khurana et al [3]	Definition: “an umbrella term broadly defined as the use of digital technologies for health” and “a means by which to increase the delivery of and access to healthcare” [3]	Domains include EHRs^c^, telehealth, mobile and wearable health technology, and artificial intelligence	A total of 40 topics across 3 subcategories (digital health knowledge, digital health skills, and digital health attitudes) were identified.
Jimenez et al [33]	Definition: “digital health refers to a broad umbrella term encompassing eHealth...broadly defined as “the use of information and communications technology in support of health and health-related fields” as well as emerging areas of advance computing sciences” [33]	Domains include eHealth, genomics, and artificial intelligence	Identified competency domains rather than competencies. Most prevalent digital health competency domains identified: electronic health/medical records, computer/tablet/app use and internet skills, practice administration/management, health information systems, and information literacy.
van Houwelingen et al [34]	Not defined: presents examples of telehealth and digital care: e-visits, devices for self-measurement, activity monitors, and personal alarms	52 competencies included for consideration: competencies focused on nursing curricula to adequately prepare nurses for the world of telehealth	32 competencies were specifically needed for telehealth provision. Competencies were identified and selected for each of the 14 nursing activities the authors included in the study.
Health Information Technology Competencies (HITComp) database [17,35]	Not defined: HITComp does not define digital health but outlines technology competencies for health care professionals	5 competency domains: administration, direct patient care, engineering/information systems/ICT^d^, informatics, and research/biomedicine	33 areas of competency are listed in the HITComp database, allowing users to select relevant areas. Competencies are defined for each domain. A total of 1025 competencies are included in the database [35].
Kinnuen et al [13]	Definition: authors quote digital health definition, “the field of knowledge and practice associated with the development and use of digital health technologies to improve health” [13,31]	Main competency domains: working in digital environment, nursing documentation, and ethics and data protection. Domains capture technologies for documenting nursing diagnosis, planned care, basic IT skills, and eHealth services	3 informatics competencies identified: ethics and data protection, nursing documentation, and digital environment.
Hübner et al [36]	Definition: defines informatics as focusing on data, information, knowledge, and user applications and defines information technology as addressing systems development and life cycle management. Health informatics described as comprised of informatics from multiple disciplines	TIGER^e^ core competencies for nursing informatics: 24 core competency areas in nursing and nursing management in health informatics clustered in 6 domains. Questionnaire used by authors included 10 technological items, including eHealth, telematics, and telehealth	The 6 domains for the TIGER competencies include “data, information, knowledge,” “information exchange and information sharing,” “ethical and legal issues,” “systems life cycle management,” “management,” and “biostatistics and medical technology.” Results showed the top 10 core competency areas for 5 different roles: clinical nursing, quality management, coordination of interprofessional care, nursing management, and IT management in nursing.

a mHealth: mobile health.

b EMR: electronic medical record.

c EHR: electronic health record.

d ICT: information and communication technology.

e TIGER: Technology Informatics Guiding Education Reform.

This state of ambiguity has resulted in an uneven and ad hoc approach to digital health education programming in undergraduate and graduate training institutions. Since the pandemic, a growing number of medical schools have implemented digital health courses, consisting mostly of electives focused on biomedical informatics or (more recently) telemedicine [3,37]. Few of these programs are integrated into the larger medicine curriculum, however [38], in part because considerable knowledge gaps remain regarding the most effective ways to integrate them [33]. Even less work has been done at the graduate level, although several novel Objective Structured Clinical Examinations (OSCEs) have been developed to provide “hands-on” training to residents [26,38-40]. Overall, systematized approaches to understanding, defining, and building digital health curriculum for medical trainees are lacking, as are those for faculty development and practicing clinicians [41].

## DDoH: A Novel Framework to Advance Digital Health Training and Competency Development

We argue that a comprehensive, multilevel approach to understanding, defining, and evaluating digital health skills for trainees is needed in order to properly prepare health professions students to meet the needs of patients in this new health care landscape. To accomplish this, we offer a model based on our growing understanding of “digital determinants of health”—the novel technological contexts and constructs that mediate an individual or community’s interactions with the health care system—and their intersections with care delivery, innovations, education, and equity.

**Figure 1.**
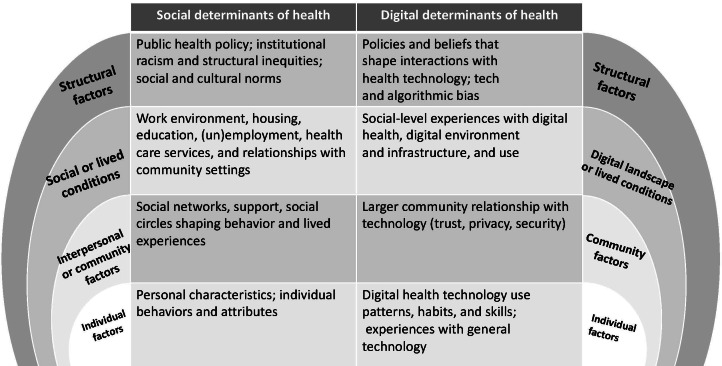
Mapping digital determinants of health to social determinants of health.

DDoH refer to antecedents within the digital environment that impact a patient’s ability to access, use, and satisfactorily experience the health care system. DDoH exist at individual, community, and structural levels [18] and include a patient’s personal experiences with digital health technology (eg, use patterns, preferences, and digital skills), communal attitudes (eg, perceptions of usefulness, trust, privacy, and surveillance), cultural beliefs and social conditions (eg, the digital environments a community experiences, including “digital deserts”), and structural factors (eg, national technology policies, bias, and discrimination; Figure 1). DDoH can act as barriers or facilitators to effective health care and may disproportionately affect certain individuals or communities [18].

The DDoH framework is modeled on the well-established social determinants of health (SDoH) framework [42]. The SDoH framework consists of conditions that shape the lived experiences of individuals and environments that impact health [43]. SDoH include both place-based conditions and “political, socioeconomic, and cultural constructs” [43]. Some examples include income level, availability of transportation, and social support and community inclusivity [43]. SDoH affect populations in negative and positive ways and can both protect health and contribute to disparities [44]. Given the developments made in understanding SDoH in the last 2 decades and integrating them in health and medicine, a large body of literature now exists exploring socioeconomic factors and the ways they shape health outcomes [45,46]. As knowledge of SDoH has expanded to health care, it has been integrated into medical curricula, and SDoH training is now considered to be a core piece in medical education [47,48]. The success of SDoH competencies and curricula development can serve as a model for integrating DDoH into medical education. The DDoH model effectively transposes SDoH thinking into digital spaces and challenges us to think beyond individual characteristics (eg, digital health literacy) when considering a person, community, or population’s interactions with digital health tools (Figure 1) [19]. DDoH are valuable to effectively conduct virtual care delivery, which is becoming more prominent in health care today [49].

## Why Teach DDoH?

Overall, we argue that digital care delivery cannot be successful or equitable without more attention to the DDoH that define it. Critical to this is the inclusion of DDoH thinking into training paradigms, programs, and resources at all levels.

Specifically, the DDoH framework can help improve digital health competency development through the following:

- *Ensures a standardized and comprehensive approach to curricular design* that would address digital health skill needs at individual, interpersonal, social, and structural levels: for example, when developing training tools to teach and evaluate a learner’s ability to assess patient “readiness” for a telemedicine appointment, educators can use the DDoH framework to include not only screening for individual digital health literacy but also an evaluation of community and social factors such as access (eg, the “digital divide”). This approach allows for a better understanding of the specific barriers to an individual patient’s use of health technologies, which can then be tailored to better meet that patient’s needs. Systematically applying these layers across learning programs creates a shared mental model for digital health training that can unify language, competency domains, and evaluation tools.
- *Can be both technology* specific *and technology* agnostic: this means that the DDoH framework can be useful when developing both specific technical skills as well as universal competencies such as patient communication, education, and shared decision-making—all of which facilitate a patient’s broader ability to successfully engage with the digital health ecosystem across devices and services. This can help avoid the ongoing challenge when developing digital health competencies, which is the tendency for technical skills to become outdated as the technologies themselves evolve (eg, computer-based web browsers vs smartphone apps vs smartwatches). For example, in teaching students EHR proficiency, using the DDoH layers can ensure that specific technical competence (eg, being able to log onto and successfully navigate the platform) is matched with interpersonal communication skills (eg, talking to the patient and *not* the EHR) and shared decision-making (eg, EHR screensharing with patients) that will serve learners regardless of the EHR platform they use.
- *Can build on existing successful SDOH-based curriculum and pedagogy,* allowing for more efficient program development and quick adaptation and validation of learning tools, rather than starting from scratch: existing SDoH programming that has been shown to be effective can quickly be adapted to DDoH contexts and tested in similar environments to assess for fidelity and effectiveness. There are now a variety of existing instructional frameworks for SDoH teaching [50] and curricula that are experiential, longitudinal, interprofessional, and community based that can be adapted [50,51]. This can also apply to preexisting SDoH evaluation tools, as well as common program requirements and other educational standards. For example, an undergraduate medical education OSCE designed to teach SDoH related to hypertension management can be quickly adapted to a case involving remote blood pressure monitoring, thereby exposing learners not only to well-known social factors related to hypertension control (eg, regular access to medications) but also unique technology-mediated factors such as access to reliable Wi-Fi for sending home values.
- *Supports digital health equity*: crucially, a DDoH-informed approach incorporates an equity-sensitive perspective into digital health training, by placing drivers of digital health disparities at the center of skills and competency development. Teaching trainees about the potential social biases of a piece of technology alongside the technology itself can help reduce the likelihood of producing clinicians that reinforce technology bias in their practice. This is particularly relevant given the growing literature exposing the relationships between digital health technology and health disparities, as well as the need for a workforce that is trained to address this even as the field expands and these tools become normalized as part of care delivery.

## Applying DDoH to Health Professions Training

Overall, health professions educators should use the DDoH framework as a guide in creating robust educational programming and evaluation tools aimed at developing health professionals who understand and can competently use digital health tools to deliver care for diverse patients. Practically, the integration of DDoH in educational programming should leverage a mixed-pedagogical approach that extends beyond passive learning and includes applied learning strategies such as OSCEs and “flipped” classrooms and innovative technologies such as virtual reality. To accomplish this effort, educators can first identify existing spaces in didactic curriculum to infuse DDoH, including adding it to SDoH training. However, a DDoH-based approach can also be taught through problem-based learning, experiential and workplace-based learning, performance assessment, and continuing medical education. Experiential “hands-on training” within community-based and service-learning opportunities (eg, homeless shelters and community advocacy organizations) can imbue technical facility while also educating on social and structural contexts of care using these technologies. In particular, connecting trainees with lay community members such as community health workers or digital navigators can expose them to the common technical skills needed to support patients as well as the social and cultural nuances of a digital health technology’s use in the real world. Incorporating trainees into health system IT efforts is another example that can provide unique administrative and regulatory contexts for learners. DDoH-sensitive learning can also complement quality improvement curricula, particularly as those programs increasingly involve EHRs, clinical decision support, informatics, and other digital health tools.

In keeping with a multilevel approach, digital health competency assessments should evaluate skills at technical, interpersonal, and structural levels, and educators applying the DDoH framework should consider stratifying their assessment domains based on these levels. For example, when developing a learning program on remote blood pressure monitoring in hypertension management, consider the following:

1. What *individual technical skills* are needed for both clinicians and patients to successfully install, set up, and transmit remote blood pressure data using currently available technologies?
2. What *immediate individual and/or community social contexts and barriers* might be relevant for patients being considered for a remote blood pressure monitoring program, and how would a clinician evaluate and address those?
3. What *larger national or structural social factors*—including scope of practice and device regulations—might impact a patient’s ability to access and use a remote blood pressure monitor, or a clinician’s ability to interpret and make medical decisions based on that data?

In this case, depending on the level of learner and training goals, while a teaching session dedicated purely to technical skill building (eg, training clinicians on the variety of remote blood pressure monitors and how to successfully take a home blood pressure measurement) may be the focus, using a DDoH-guided approach would allow educators to increase the value of the training by teaching clinicians to also address individual DDoH needs (eg, language preferences and physical or cognitive accessibility needs) and social layers (eg, local library Wi-Fi access) that may contribute to a blood pressure device’s ultimately successful use.

There are some challenges to creating an educational system based on the DDoH framework. Designing robust experiences is time-consuming and often labor-intensive. Consequently, it is important to identify already developed programs that can be quickly adapted and evaluated; this can include existing SDoH programs, but it can also include the myriad of ad hoc telemedicine training tools that proliferated during the COVID -19 pandemic that can now be reworked to be more robust and structured. In general, flexible learning approaches that can respond to the short technology life cycles of many of these products are critical to ensure that skills remain relevant or can be quickly updated; this is challenging to keep on top of and may favor a longitudinal approach that offers multiple teaching touch points throughout a training program. Finding competent faculty to teach these skills may also be difficult, as practicing clinicians and educators are often learning about novel technologies alongside trainees. Finally, convincing health care stakeholders that DDoH are worth studying, learning, and evaluating in their own right take efforts, particularly given the other demands and competing priorities of health training. However, we strongly believe these technologies will only continue to proliferate and become further embedded in health care delivery, and ignoring their outsized and disruptive role in clinical care in critical training periods is ultimately a disservice to the health care workforce and patients.

## Summary

There is growing need to develop unified digital health education and training competencies for health professions students. Efforts to cultivate a workforce adept in digital health tools must prioritize understanding and mitigating the digital determinants of health that shape individuals’ interactions with health care technology. Using a DDoH framework in medical education—including not only didactic training but also hands-on skill building, as well as continuing education opportunities—can help guide robust educational programming and evaluation tools aimed at developing health professionals who understand and can competently use digital health tools to deliver care for diverse patients.

## Abbreviations

DDoH: digital determinants of health
EHR: electronic health record
OSCE: Objective Structured Clinical Examination
SDoH: social determinants of health
